# Breast adenomyoepithelioma: a case report with malignant proliferation of epithelial and myoepithelial elements

**DOI:** 10.1186/1477-7819-11-285

**Published:** 2013-10-30

**Authors:** Vincenzo Petrozza, Giulia Pasciuti, Alberto Pacchiarotti, Federica Tomao, Federica Zoratto, Luigi Rossi, Antonella Fontana, Fabiana Censi, Barbara Sardella, Claudio Di Cristofano, Natale Porta, Carlo Della Rocca

**Affiliations:** 1 Department of Medical-Surgical Sciences and Biotechnologies, Histopathology Unit, Polo Pontino - Sapienza University of Rome, Latina, Italy; 2 Department of Medico-Surgical Sciences and Biotechnologies, Oncology Unit, S. Maria Goretti, Hospital - Sapienza University of Rome, Latina, Italy; 3 San Marco Clinic, Pathology Unit, Latina, Italy; 4 Department of Obstetrics and Gynecology, Sapienza University of Rome, Rome, Italy; 5 Department of Radiotherapy, S. Maria Goretti Hospital, Latina, Italy; 6 Department of Pathology, S. Maria Goretti Hospital, Latina, Italy

**Keywords:** Breast cancer, Malignant adenomyoepithelioma, Immunohistochemical

## Abstract

**Background:**

Breast adenomyoepithelioma is an unusual tumor characterized by a biphasic proliferation of epithelial and myoepithelial cells. Most breast adenomyoepitheliomas are considered to be benign or to have a low-grade malignant potential, characterized by propensity for local recurrence. Malignant changes arising in this lesion are extremely rare and may involve one or both cellular components.

**Case report:**

We discuss a case of a 60 year-old woman who began to experience pain in her right breast in January 2009. Breast ultrasound and mammography were performed showing a rounded, hypoechoic solid lesion with ill-defined margins in the right inner-inferior quadrant, suspicious of malignancy. Quadrantectomy of the inner-inferior quadrant of the right breast with sampling of ipsilateral axillary lymph nodes was performed. The histological analysis confirmed the diagnosis of adenomyoepithelioma with focal malignant change of the epithelial component, associated with high-grade malignant myoepithelial change. The patient was treated with adjuvant radiotherapy and her right breast received a dose of Gy 50 with a boost of Gy 10 to the tumor bed. At present, the patient shows no sign of tumor recurrence.

**Conclusion:**

Breast malignant adenomyoepithelioma is a rare tumor which should be considered in the differential diagnosis of other solid breast lesions. Only few cases have been reported in the literature. Diagnosis, optimal therapy and predicting the outcome are problematic issues due to the rarity of this disease which appears to have hematogenous rather than lymphatic spread and usually occurs in primary tumors ≥ 1.6 cm in size.

## Background

Proliferative lesions of the mammary gland arise mainly in the terminal duct lobular unit (TDLU). Most lesions exhibit epithelial alterations; however, some mammary lesions show myoepithelial changes [1]. Myoepithelial basal cells are localized between luminal epithelial cells and the stroma, which place them in an ideal position in order to communicate with both compartments. The myoepithelial cells express typical cytokeratins of the basal layer of stratified epithelia (CK5, CK14, and CK17), filamentous *α*-smooth muscle actin (SMA) and the heavy chain-myosin (hc-myosin). Some tumor suppressor proteins, including p63, p73, 14-3-3 sigma, maspin and Wilms Tumor (WT-1) have been preferentially detected in myoepithelial cells [2]. Recent data indicate the existence of a morphologically distinct myoepithelial cell, which lacks expression of SMA and in some cases also lacks CK5, CK14, and CK17 [3], indicating that a hierarchical differentiation pattern may exist within the myoepithelial lineage. Myoepithelial cells rarely transform; however, when they do transform, they generally give rise to benign or low-grade malignant tumors. The lesions which show a myoepithelial component are rare in the mammary gland, and they are often benign and biphasic, with an epithelial and a myoepithelial basal component [1]. The adenomyoepithelioma is a biphasic tumor, either benign or with low potential of malignancy, which can be found in salivary gland, skin adnexal and lung, but is more frequent in the mammary gland. This entity rarely progresses to a more malignant state or gives rise to metastasis [1]. Morphological features of malignancy that could predict the potential for local recurrence and/or metastasis are not well-established. The likelihood of recurrence seems to be associated with incomplete removal [4], cellular pleomorphism, mitoses, necrosis, invasion of the surrounding tissue and association with other types of malignant tumors such as invasive ductal carcinoma and undifferentiated carcinoma [5]. The classification of the World Health Organization (WHO, 2012) divides the adenomyoepithelioma into a benign type where both the epithelial and myoepithelial component are histologically non-malignant and a form which shows a malignant transformation [6]. Although rare, this malignant transformation can develop from one of the two components, epithelial and myoepithelial, or from both. This case of malignant adenomyoepithelioma is of interest not only for its rarity, but also for the peculiar aspects of the malignant component that in some areas shows morphological and immunophenotypical similarities to basal-like breast carcinoma.

## Case presentation

**Figure 1:**
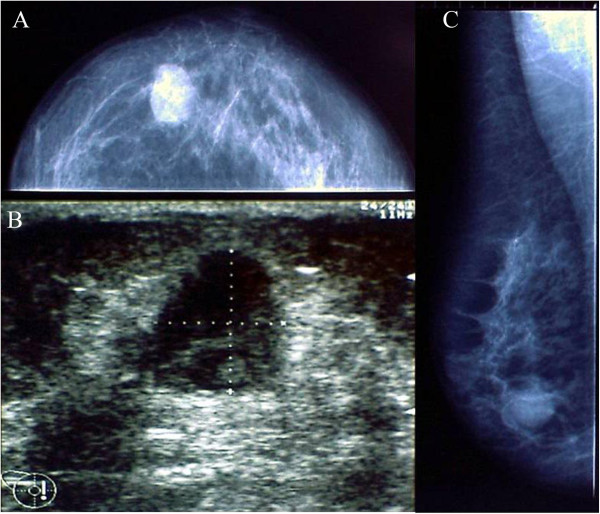
Radiological characteristics of lesion; (A and B) mammography craniocaudal and mediolateral oblique views of the right breast show an ill-defined end opacity in the inner-inferior quadrant of about 20 mm diameter; (C): ultrasonography of the right breast shows a hypoechoic nodule of about 17 mm diameter around the right mammary region near the areola.

**Figure 2:**
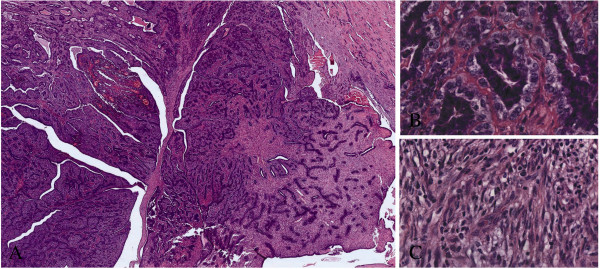
Morphological characteristics of the lesion; (A) the lesion shows multilobulated outlines and pushing margins (hematoxylin-eosin, magnification 4×); (B) the benign component is composed of rounded or elongated tubules lined by a single layer of epithelial cells and one or two layers of myoepithelial cells (hematoxylin-eosin, magnification 40); (C) the malignant component shows cytological atypia of epithelial and myoepithelial components (hematoxylin-eosin, magnification 40×).

**Figure 3:**
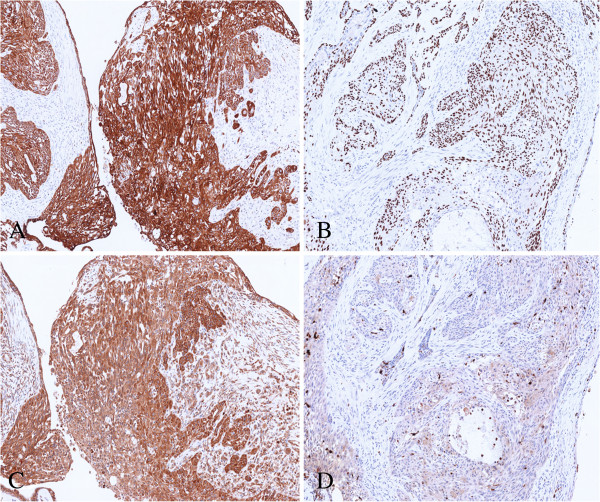
Immunohistochemical characteristics of the lesion; (A) expression of CK 7 (magnification 10×); (B) expression of p63 (magnification 10×); (C) expression of CK 5 (magnification 10×); (D) expression of S-100 (magnification 10×).

We describe a 60 year-old female patients, of Caucasian origin, in generally good health (PS = 0), with no family history of cancer. In January 2009, the patient developed pain in the right mammary region. The onset of this pain was initially attributed to a trauma she had had in this area. The patient underwent several clinical examinations which included echography of both mammary glands and corresponding axillary cavities. The ultrasonography highlighted an oval shaped hypoechoic mass of about 17 mm around the right mammary region near the areola, on the border between superior and internal-inferior quadrants (Figure 1). The mammography confirmed the presence of this nodular lesion which was about 20 mm (Figure 1). After the radiological examinations, a fine needle aspiration of the lesion was performed. The sample taken was not diagnostic. After surgical *videat*, lumpectomy around the areola was performed on the patient. The histological analysis of the mammary lumpectomy revealed an adenomyoepithelioma with focal malignant transformation of the epithelial component (2.4 mm) and a more spread malignant transformation of the myoepithelial component (12 mm) without necrosis and metastasis in the lymph nodes (Figures 2 and 3). The surgical margin was free (distance over 2 cm). For the clinical staging, the patient underwent a chest X-ray, echography of the liver and a bone scan. All the examinations were negative. The right mammary gland of the patient was treated with radiotherapy (gray (Gy) 50 total dose plus a boost of Gy 10 to the tumor bed). At present, the patient shows no signs of tumor recurrence. The histological report describes a lesion with multilobulated outlines, pushing margins and biphasic aspects due to the presence of a double population of cells. The lesion is composed of epithelioid elements with an oval-shaped nucleus, minute nucleolus and eosinophilic cytoplasm with sharp margins (epithelial-like component). These cells are arranged in glandular-like structures, distorted and compressed by elongated cells with clear cytoplasm (myoepithelial-like component). This second population of cells is more abundant in number. The epithelial-like component shows an average proliferative index of three mitoses for ten high power fields. However, there is a focal area (in the section) of 2.4 mm of highly atypical epithelioid cells arranged in sheets and clumps with features of stromal infiltration. In this area, the epithelioid cells show an average proliferative index of seven mitoses for ten high power fields. The myoepithelial-like component, present in larger quantities, shows in part (12 mm in diameter) histologically malignant aspects due to the presence of atypical cells and an average proliferative index of 32 mitoses for 10 high power fields. An immunohistochemical study was then carried out using the following antibodies: cytokeratin 5 (clone XM26 Novocastra Leica Biosystems), cytokeratin 6 (clone LHK6B Novocastra Leica Biosystems), cytokeratin 7 (clone OV-TL 12/30 Novocastra Leica Biosystems), cytokeratin 8/18 (clone 5D3 Novocastra Leica Biosystems), cytokeratin 14 (clone LL002 Novocastra Leica Biosystems), cytokeratin 19 (clone B170 Novocastra Leica Biosystems), estrogen receptors (clone 6F11 Novocastra Leica Biosystems), progesterone receptors (clone 16 Novocastra Leica Biosystems), HER-2 (HercepTest DAKO), p53 (clone DO7 Novocastra Leica Biosystems), p63 (clone 7JUL Novocastra Leica Biosystems), CD10 (clone 56C6 Novocastra Leica Biosystems), e-cadherin (clone 36B5 Novocastra Leica Biosystems), vimentin (clone V9 Novocastra Leica Biosystems) and S100 (clone S100 Novocastra Leica Biosystems). The immunohistochemical reactions were evaluated with a semiquantitative method. The cellular proliferative index was evaluated by Ki-67 (clone MM1 Novocastra) and showed 10% of nuclear cellular positivity. The immunohistochemical analysis showed the epithelial luminal nature of the epithelioid benign component because this component was positive for luminal cytokeratins (7, 8/18, 19) (Table 1). The malignant epithelioid component of the lesion was largely positive for luminal cytokeratin 7 and locally positive for myoepithelial-basal markers (p63, S100, cytokeratins 5, 6, 14) (Table 1). The elongated benign and malignant cells of the lesion gave a positive result with all myoepithelial-basal markers (Table 1).

**Table 1:** Immunohistochemical results

	**VIM**	**CK5**	**CK6**	**CK7**	**CK8/18**	**CK14**	**CK19**	**P63**	**S100**	**CD10**	**ER**	**PG**	**HER-2**
EM	**-**	**−/+**	**+**	**+**	**-**	**+**	**-**	**−/+**	**−/+**	**-**	**-−/+**	**-**	**-**
EB	**-**	**−/+**	**−/+**	**+**	**−/+**	**+**	**−/+**	**-**	**-**	**-**	**−/+**	**−/+**	**−/+**
MM	**+**	**-**	**+**	**−/+**	**-**	**-**	**-**	**+**	**+**	**+**	**-−/+**	**-**	**-**
MB	**+**	**−/+**	**+**	**−/+**	**-**	**-**	**-**	**+**	**+**	**+**	**-−/+**	**-**	**-**

Legend:

EM, epithelial malignant component.

EB, epithelial benign component.

MM, myoepithelial malignant component.

MB myoepithelial benign component.

negative (−) antibody was not found in any cell.

largely positive (+) > 75% of the cells were positive.

locally positive (−/+) 25 to 75% of the cells were positive.

focally positive (−/+) < 25% of the cells were positive.

## Discussion

The adenomyoepithelioma of the breast is uncommon, often benign or with low potential of malignancy or local recurrence. Metastases arising from malignant variants of such tumors also occur rarely although several such cases have been reported [7]. Review of the literature reveals that malignant lesions appear to fall into two broad groups: those that are readily identified as consisting of areas of obvious malignancy arising in conjunction with an otherwise typical adenomyoepithelioma [1] and those with the overall appearance of an adenomyoepithelioma but seen on close examination to contain foci of cellular atypia and increased mitotic activity. Recurrent tumors and distant metastases have been reported in association of both groups. Among the 11 cases of either epithelial and myoepithelial changes included in these studies [7,8], two cases showed malignant transformation of the myoepithelial component only [1,9] and one cases showed malignant transformation of the epithelial component only [10] (Table 2). Because of the capacity of adenomyoepithelioma with both epithelial and myoepithelial malignant transformation to metastasize, this entity should preferably be treated by wide local excision with appropriate margins [7]. The presence of metastases was described in patients with a tumor of ≥ about 1.6 cm. The metastases spread mainly via the blood system, with brain and lungs as main target organs. However, there are no data about sentinel lymph node removal and/or lymphadenectomy and the adjuvant radiotherapy and/or chemotherapy. The genesis of infiltrating breast cancer today is considered to be a complex process with several phases that originates from stem cells of the terminal duct lobular unit (TDLU) [11]. The identification of adult stem cells in the mammary tissue in the TDLU, that are able to differentiate themselves initially into epithelial glandular precursor cells (CK5 and CK8/18 positive), or into precursor cells of the myoepithelial layer (CK5 and SMA positive) and, subsequently, into mature luminal cells (CK8/18 positive) and mature myoepithelial cells [12,13] (SMA positive), confirmed the distinction of the breast cancer in luminal, basal, HER-2 positive and ‘similar to normal’ breast highlighted by gene expression studies [14,15]. Nielsen *et al*. proposed a panel composed of immunohistochemical markers anti-ER, anti-EGFR, anti-HER2, and anti-CK 5/6 to identify basal breast cancer [16]. In our case, the epithelioid malignant component revealed a widespread expression of the epithelial luminal CK7 marker together with expression of the ‘staminal’ CK5 marker and the myoepithelial p63 and S-100 markers which could suggest that the origin of this component is from the stem cells of the mammary gland with an intermediate epithelial-mesenchymal differentiation. Reis-Filho *et al*. showed a basal-like immunophenotype (ER-, HER2-, EGFR + and/or CK5/6 +) in 59 out of 65 cases of metaplastic breast cancer that produces stromal matrix that is an epithelial carcinoma with areas of mesenchymal differentiation [17]. Futhermore, a case of malignant adenomyoepithelioma of the breast that produces stromal matrix was described with immunohistochemical profile of myoepithelial differentiation (S100 e maspin positive) but not basal-like (CK14-negative) in the metaplastic component [18]. The presence of neoplastic components with an intermediate epithelial-mesenchymal differentiation in biphasic tumors of mammary gland suggests that these lesions can originate from mammarian stem cells with the potential to differentiate themselves into epithelial or into mesenchymal-myoepithelial cells.

**Table 2:** Malignant epithelial and myoepithelial adenomyoepithelioma of the breast with metastases: meta-analysis

**First author**	**Age (years)/sex**	**Size (cm)**	**Early treatment**	**Metastasis after early treatment**
Bult [4]	52/F	1.6	Radical modified mastectomy	12Y thyroid
Kihara [19]	86/F	4.0 × 3.5 × 3.5	Simplex mastectomy with lymphoadenectomy	3M lung
Loose [20]	42/F	3.5 × 3 × 2	Excisional biopsy	52M local recurrences
				54M lung
				60M brain
Nadelman [8]	46/F	not described	Lumpectomy	1Y lung
				2Y local recurrences
				3Y local recurrences
Nadelman [8]	73/F	not described	Not performed	S lung
Rasbridge [21]	76/F	15	Extensive local excision	36M brain
Takahashi [22]	60/F	9 × 8 × 6	Radical mastectomy	24M lung, bone, mediastinal lymph nodes
Trojani [23]	51/F	2	Excisional biopsy	2Y lung
Chen [24]	47/F	17 × 11 × 6	Radical modified mastectomy	3W bone
Jones [9]	71/F	3 × 3 × 2	Mastectomy with lymphadenectomy	2Y liver on autopsy
Foschini [10]	60/F	4	Lumpectomy	21M lung

Legend: F, female; Y, years; M, months; W, weeks.

## Conclusion

Breast malignant adenomyoepithelioma is a rare tumor which should be considered in the differential diagnosis of other solid breast lesions. Only a few cases have been reported in the literature and establishing the diagnosis, determining the optimal therapy as well as predicting the outcome are problematic issues due to the rarity of this disease. The adenomyoepithelioma appears to have hematogenous rather than lymphatic spread and usually occurs in primary tumors ≥ 1.6 cm in size.

## Consent

Written informed consent was obtained from the patient for publication of this case report and any accompanying images. A copy of the written consent is available for review by the Editor-in-Chief of this journal.

## Abbreviations

CK: Cytokeratins; EB: Epithelial benign component; EM: Epithelial malignant component; Gy: Gray; MB: Myoepithelial benign component; MM: Myoepithelial malignant component; PS: Performance status; SMA: Smooth muscle actin; TDLU: Terminal duct lobular unit; WHO: World Health Organization.

## Competing interests

The authors declare that they have no competing interests.

## Authors’ contributions

VP have made substantial contributions to conception and design. GP have made substantial contributions to acquisition of data. AP have made substantial contributions to conception and design. FT have made substantial contributions to acquisition of data. FZ have made substantial contributions to acquisition of data. LR have made substantial contributions to acquisition of data. AF have made substantial contributions to acquisition of data. FC have been involved in drafting the manuscript. BS have been involved in drafting the manuscript. CDiC agree to be accountable for all aspects of the work in ensuring that questions related to the accuracy or integrity of any part of the work are appropriately investigated and resolved. NP have been involved in drafting the manuscript and revising it critically for important intellectual content. CDR have given final approval of the version to be published. All authors read and approved the final manuscript.
